# DHA brain uptake and *APOE4* status: a PET study with [1-^11^C]-DHA

**DOI:** 10.1186/s13195-017-0250-1

**Published:** 2017-03-23

**Authors:** Hussein N. Yassine, Etienne Croteau, Varun Rawat, Joseph R. Hibbeln, Stanley I. Rapoport, Stephen C. Cunnane, John C. Umhau

**Affiliations:** 1 Department of Medicine, University of Southern California, 2250 Alcazar Street, Room 210, Los Angeles, CA 90033 USA; 2 Research Center on Aging, University of Sherbrooke, Sherbrooke, QC Canada; 3 Section on Nutritional Neurosciences, National Institute on Alcohol Abuse and Alcoholism, National Institutes of Health, Rockville, MD USA; 4 Brain Physiology and Metabolism Section, National Institute on Aging, National Institutes of Health, Bethesda, MD USA; 5 Division of Psychiatry Products, Center for Drug Evaluation and Research, U.S. Food and Drug Administration, College Park, MD USA

**Keywords:** APOE, Alzheimer’s disease, DHA, PET

## Abstract

**Background:**

The apolipoprotein E ɛ4 (*APOE4*) allele is the strongest genetic risk factor identified for developing Alzheimer’s disease (AD). Among brain lipids, alteration in the ω-3 polyunsaturated fatty acid docosahexaenoic acid (DHA) homeostasis is implicated in AD pathogenesis. *APOE4* may influence both brain DHA metabolism and cognitive outcomes.

**Methods:**

Using positron emission tomography, regional incorporation coefficients (k*), rates of DHA incorporation from plasma into the brain using [1-^11^C]-DHA (*J*_in_), and regional cerebral blood flow using [^15^O]-water were measured in 22 middle-aged healthy adults (mean age 35 years, range 19–65 years). Data were partially volume error-corrected for brain atrophy. APOE4 phenotype was determined by protein expression, and unesterified DHA concentrations were quantified in plasma. An exploratory post hoc analysis of the effect of *APOE4* on DHA brain kinetics was performed.

**Results:**

The mean global gray matter DHA incorporation coefficient, k*, was significantly higher (16%) among *APOE4* carriers (*n* = 9) than among noncarriers (*n* = 13, *p* = 0.046). Higher DHA incorporation coefficients were observed in several brain regions, particularly in the entorhinal subregion, an area affected early in AD pathogenesis. Cerebral blood flow, unesterified plasma DHA, and whole brain DHA incorporation rate (*J*_in_) did not differ significantly between the *APOE* groups.

**Conclusions:**

Our findings suggest an increase in the DHA incorporation coefficient in several brain regions in *APOE4* carriers. These findings may contribute to understanding how *APOE4* genotypes affect AD risk.

## Background

Apolipoprotein E ɛ4 (*APOE4*) genotype is the strongest genetic risk factor for late-onset or sporadic Alzheimer’s disease (AD). APOE proteins, the product of the *APOE* gene, have isoform-specific functions. For example, APOE’s affinity for the low-density lipoprotein receptor is known to differ between isoforms (APOE4 > APOE3 > APOE2) [1]. These differences have implications for the metabolism of APOE lipoprotein particles and the amount of lipid carried by APOE. In the brain, APOE forms high-density lipoprotein particles and participates in exchange of lipids between glial cells and neurons [2]. Clinical and animal studies indicate that brain APOE particle size and number differ by *APOE* genotype [3–5]. In plasma, APOE4 is catabolized faster with a plasma residence time of approximately half that of APOE3 [6].

Among brain lipids, the ω-3 polyunsaturated fatty acid (PUFA) docosahexaenoic acid (DHA, 22:6ω-3) may be of particular importance in AD pathogenesis. DHA forms up to 40% of fatty acids in certain gray matter lipids and is concentrated at synapses, where it plays a role in synaptic plasticity [7]. In embryonic neuronal cultures, DHA supplementation promotes neurite growth and synaptic protein expression [8]. Severe long-term dietary deficiency of DHA leads to learning impairment in animal models [9]. The brain also requires DHA for maintenance of neuronal membranes, production and clearance of β-amyloid 42, modulation of inflammation [10, 11], and cerebrovascular health [12]. We previously reported a direct association between lower serum DHA levels and greater cerebral amyloidosis in cognitively healthy older adults [13]. The lowest quartile of serum DHA was associated with significantly greater cerebral amyloid deposition, smaller entorhinal and hippocampal volumes, and worse nonverbal memory scores [13].

DHA’s incorporation into the brain can be assessed by positron emission tomography (PET) following intravenous infusion of carbon-11 ([1-^11^C])-DHA using the incorporation coefficient k* [14]. k* represents multiple steps, including DHA diffusion from plasma to brain cells, intracellular DHA acylation to DHA-CoA by an acyl-coenzyme A (acyl-CoA) synthetase, and DHA transfer from DHA-CoA to membrane lysophospholipids by an acyltransferase [15]. k* is independent of changes in regional cerebral blood flow (rCBF). For example, rCBF can be doubled using CO_2_ inhalation without changing k* [16]. The net rate of DHA incorporation from plasma (*J*_in_) is the product of unesterified plasma DHA times k*. At steady state, *J*_in_ is equivalent to the net loss of DHA from the brain (*J*_out_). Chronic dietary ω-3 PUFA deprivation leads to increased k* in the face of a 40-fold reduction in the rate of DHA loss (*J*_out_) from the brain [17].

*APOE* genotype may influence the metabolism of DHA in the brain or its delivery to the brain, although brain DHA delivery may not directly depend on peripheral lipoproteins [18]. In humans, whole body DHA half-life was lower in *APOE4* carriers than in noncarriers, which was attributed to greater liver oxidation of DHA [19]. Brain DHA levels were lower in older but not younger *APOE4* targeted replacement (TR) mice than in age-matched *APOE2* TR mice [20]. We found lower cerebrospinal fluid (CSF) DHA levels in older *APOE4* carriers with mild AD after 18 months of DHA supplementation than in *APOE4* noncarriers [21]. The goal of the present study was to explore the effect of *APOE4* on [1-^11^C]-DHA brain kinetics in a group of 22 healthy adults using PET.

## Methods

### Participants

We obtained plasma samples from 22 healthy control subjects between 19 and 65 years of age to assess APOE4 expression and APOE plasma levels. These subjects were recruited from the Bethesda, MD, USA, area [22]. The present report describes results from the control arm only of an alcohol withdrawal study. Participants were nonsmokers and reported no medication, drug, or alcohol use for at least 2 weeks prior to the PET scan. All participants underwent an extensive history and physical examination with laboratory tests to ensure that they were free of significant medical problems and had no history of neurological or psychiatric disorders. Three days preceding the PET scan, participants were instructed to avoid foods high in ω-3 PUFAs (e.g., seafood). The Diet History Questionnaire was used to assess dietary habits 12 months preceding the study [23].

### PET imaging

The PET protocol involved first injecting a bolus of [^15^O]-water to image rCBF. PET scans were acquired at approximately 11:00 a.m. following 24 h on a standardized low-DHA diet and an overnight fast. Blood was collected three times during the scan to quantify plasma unesterified fatty acid concentrations and tracer radioactivity. Fifteen minutes following the injection of [^15^O]-water, 1118 MBq of [1-^11^C]-DHA was infused intravenously for 3 minutes at a constant rate (Harvard Infusion Pump, South Natick, MA, USA). Because of the high specific activity of [1-^11^C]-DHA, less than 0.06 mmol of unlabeled DHA was infused into a subject, so there was no significant pharmacological or tracee effect of the dose of the tracer itself. Serial dynamic three-dimensional scans were acquired during the hour following the start of the infusion. Arterial samples (2–5 ml) were obtained at fixed times to determine radioactivity in whole blood and plasma.

### Input function

To rapidly assay plasma [1-^11^C]-DHA during a PET scan, a solid-phase extraction procedure to separate unesterified [1-^11^C]-DHA from remaining plasma radioactivity was used. From plasma samples collected at 0, 3, 7, 10, 15, 20, 40, and 60 minutes after infusion of [1-^11^C]-DHA, total lipids were extracted into chloroform:methanol (1:1) as previously described [24].

### Coregistration of PET scans to brain anatomy

Magnetic resonance imaging (MRI) scans of the brain were obtained with a 1.5-Tesla Horizon scanner (GE Medical Systems, Milwaukee, WI, USA). This produced T1-weighted volumetric spoiled gradient MRI scans for superimposition onto the PET images and to register both rCBF images from the [^15^O]-water scans and [1-^11^C]-DHA parametric images. Appropriate coregistration of the PET images onto the MRI studies was visually verified for each participant. Because of the poor spatial resolution of a PET scan, underestimation of higher radioactivity can occur in gray matter regions. To provide the most accurate measure of radioactivity in specific gray matter regions, partial volume error (PVE) was corrected. PVE correction is particularly important when studying disorders associated with cerebral atrophy, such as aging, cognitive decline, and AD. It provides a better measure of actual tissue metabolism or blood flow free of effects of CSF, and it corrects for loss (spill-out) of the radioactive signal to adjacent tissue and for spill-in of signal from adjacent tissue.

### Regions of interest

**Fig. 1:**
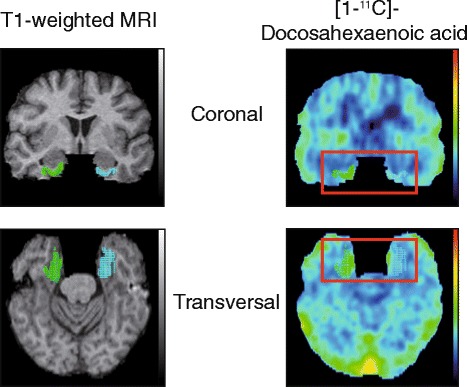
FreeSurfer segmentation (*green* = right hemisphere, *blue* = left hemisphere) of the entorhinal region of interest superimposed on a sum image (60 minutes) of [^11^C]-docosahexaenoic acid ([1-^11^C]-DHA) and T1-weighted magnetic resonance imaging scans of one participant. DHA incoporation coefficient k* in the entorhinal cortex is illustrated in the *red rectangles*

Following injection of [1-^11^C]-DHA, k* (μl/minute/ml) was calculated from the PVE-corrected PET brain images using a one-tissue compartment model as described previously [24]. Two approaches were used to perform the image analysis. First, regions of interest (ROIs) were drawn manually on individual MRI scans on six continuous axial MRI slices at the National Institutes of Health PET center [24]. PVE-corrected values of k* and rCBF were obtained for all regions from PET images by limiting averaging to voxels identified as gray matter by the segmentation procedure. Second, T1-weighted MRI FreeSurfer segmentation was used for the kinetic analysis of ROIs of the [1-^11^C]-DHA cerebral dynamic acquisitions from 21 of the 22 participants at the University of Sherbrooke, Sherbrooke, QC, Canada. Figure 1 presents an illustration of [1-^11^C]-DHA k* focused in the entorhinal cortex area of one of the participants.

### APOE phenotyping and ApoE plasma levels

APOE4 phenotype was obtained by Western blotting of plasma samples using a previously validated APOE4-specific antibody (8941S; Cell Signaling Technology, Danvers, MA, USA). The validity of the antibody was confirmed using samples of known *APOE* genotype. *APOE4* status was defined by visible APOE4 bands after Western blotting of plasma samples. APOE plasma levels were measured using an in-house enzyme-linked immunosorbent assay with inter- and intraassay coefficients of variation <10% [25].

### Data analysis

Data are presented as means with SDs. The two *APOE* groups were compared using an independent *t* test or linear regression modeling to adjust for covariates. Age and sex were added to the linear model as covariates with k* as the dependent variable and *APOE* group as the independent variable. The variables were correlated using Pearson’s correlations for normally distributed data or Spearman’s correlations for nonnormally distributed data. Within the brain regions, we focused on the medial temporal lobe subregions, given their significance in AD, with FreeSurfer segmentation to assess ROI [1-^11^C]-DHA kinetics. *p* ≤ 0.05 was considered a significant difference.

## Results

### Participant characteristics

Our study sample included 13 *APOE4*-negative and 9 *APOE4*-positive participants, based on the detection of APOE4 proteins in plasma by Western blotting. The participants were mostly middle-aged white individuals who were not obese and were without diabetes or dyslipidemia. The participants’ characteristics did not differ by *APOE* genotype. Additional characteristics and biochemical measurements are presented in Table 1.

**Table 1:** Participant characteristics

Group	*APOE4* noncarriers (*n* = 13)	*APOE4* carriers (*n* = 9)	*p* Value
Female/male sex	8/5	2/7	0.07
White/nonwhite race	8/5	4/5	0.93
Age, years	37.1 (15.7)	32.1 (10.7)	0.39
Weight, kg	77.1 (15.7)	85.7 (20.4)	0.30
BMI, kg/m^2^	26.2 (4.2)	27.1 (5.0)	0.64
Systolic blood pressure, mmHg	111 (9.2)	118.4 (14.7)	0.35
Diastolic blood pressure, mmHg	60.3 (10.6)	62.8 (7.2)	0.61
Fasting glucose, mg/dl	94 (9.3)	90 (9.5)	0.37
Total cholesterol, mg/dl	162 (42.9)	168.1 (31.1)	0.70
HDL-C, mg/dl	49.8 (17.7)	53.7 (11.9)	0.54
LDL-C, mg/dl	98.3 (33.4)	101 (28.8)	0.84
Estimated DHA intake based on DHQ, mg/day	40 (41)	130 (120)	0.1
Brain volume, ml	1246 (150)	1232 (62)	0.76
Plasma APOE levels, μg/ml	15.8 (5.7)	10.7 (6.1)	0.06

*Abbreviations: APOE* Apolipoprotein E, *APOE4* Apolipoprotein E ɛ4, *BMI* Body mass index, *DHA* Docosahexaenoic acid, *DHQ* Diet History Questionnaire, *HDL-C* High-density lipoprotein cholesterol, *LDL-C* Low-density lipoprotein cholesterol

Values are presented as mean (SD). Groups were compared using an independent t test

### DHA incorporation coefficient, k*

**Fig. 2:**
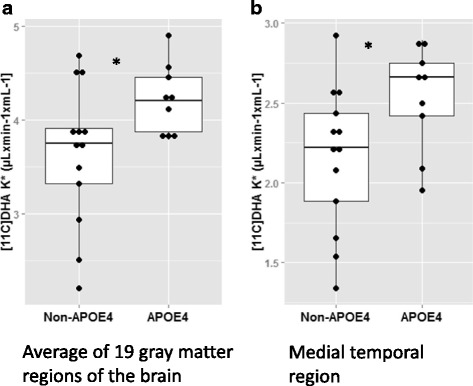
Mean global brain (**a**) and medial temporal lobe (**b**) docosahexaenoic acid (DHA) incorporation coefficient k* by apolipoprotein E gene (*APOE*) subgroups. Significantly greater DHA uptake was observed in *APOE4* carriers than in noncarriers (*p ≤ 0.05)

The mean global gray matter k* was 16% higher in *APOE4* carriers than in noncarriers (*p* = 0.04) (Fig. 2a). k* was significantly higher in several gray matter subregions (Table 2), but it did not differ in the white matter by *APOE* subgroup. Age did not correlate with k* (*r* = −0.006, *p* = 0.9), but only 4 of the 23 participants were older than 50 years of age. BMI (*r* = −0.04, *p* = 0.89) and sex (*p* = 0.8) also did not correlate with k*. Including age (*p* = 0.12) or sex (*p* = 0.1) attenuated the effect of *APOE* on global gray matter k*. The k* in the medial temporal lobe was 17% higher in *APOE4* carriers than in noncarriers (*p* = 0.035) (Fig. 2b). On the basis of FreeSurfer segmentation for the kinetic analysis of ROI of the [1-^11^C]-DHA dynamic acquisitions in the medial temporal lobe, the most pronounced difference in k* was observed in the right entorhinal region (34% greater in *APOE4* carriers than in noncarriers; *p* = 0.05) (Table 3). A significant inverse correlation was observed between k* and blood volume in the medial temporal lobe (*r* = −0.42, *p* = 0.05).

**Table 2:** Regional DHA k* by *APOE* subgroup (μl∙minute^−1^∙ml^−1^)

Regions	*APOE4* noncarriers (*n* = 13)	*APOE4* carriers (*n* = 9)	*p* Value	Adjusted *p* value^a^
Orbitofrontal	4.84 (1.25)	5.20 (0.59)	0.42	0.79
Prefrontal	3.81 (0.76)	4.37 (0.56)	0.07	0.12
Premotor	3.86 (0.73)	4.65 (0.51)	0.01	0.057
Anterior cingulate	2.76 (0.55)	3.27 (0.31)	0.02	0.088
Inferior temporal	3.56 (0.87)	3.96 (0.37)	0.21	0.56
Middle temporal	3.72 (0.83)	4.11 (0.39)	0.21	0.57
Superior temporal	3.42 (0.88)	3.69 (0.50)	0.41	0.87
Medial temporal	2.16 (0.45)	2.53 (0.33)	0.046	0.28
Sensorimotor	3.95 (0.84)	4.78 (0.52)	0.016	0.12
Inferior parietal	3.89 (0.87)	4.42 (0.46)	0.26	0.40
Superior parietal	4.38 (1.09)	4.81 (0.35)	0.26	0.59
Medial parietal	3.55 (0.88)	4.35 (0.38)	0.018	0.087
**Posterior cingulate**	**3.09 (0.71)**	**4.00 (0.34)**	**0.002**	**0.02**
Occipital association	3.98 (0.86)	4.81 (0.47)	0.016	0.12
Calcarine	4.41 (1.01)	5.07 (0.64)	0.098	0.35
Thalamus	3.90 (0.99)	4.62 (0.43)	0.053	0.06
Striatum	3.57 (0.95)	4.16 (0.84)	0.149	0.11
Cerebellar hemisphere	3.30 (0.82)	3.91 (0.53)	0.141	0.29
Vermis	2.97 (0.81)	3.60 (0.50)	0.051	0.046
Pure white matter	1.09 (0.41)	1.26 (0.16)	0.25	0.21
Average gray matter	3.64 (0.75)	4.23 (0.37)	0.04	0.19

*APOE4* Apolipoprotein E ɛ4

Data are presented as mean (SD). Docosahexaenoic acid k* values were partial volume error-corrected. Groups were compared using a linear regression model. Significantly differences (*p* < 0.05) by *APOE* group are shown in bold type

^a^ Adjusted to age and sex

**Table 3:** Docosahexaenoic acid incorporation coefficient (k*, μl∙minute^−1^∙ml^−1^) in medial temporal lobe subregions according to *APOE* genotype

	*APOE4* noncarriers (*n* = 12)	*APOE4* carriers (*n* = 9)	*p* Value
Hippocampus	2.69 (0.73)	2.86 (0.35)	0.43
Left	2.8 (0.80)	2.9 (0.4)	0.60
Right	2.6 (0.70)	2.8 (0.4)	0.47
Entorhinal cortex	2.77(0.10)	3.57 (0.67)	0.056
Left	2.6 (1.10)	3.2 (0.5)	0.16
Right	2.9 (1.0)	3.9 (1.2)	0.05

*APOE* Apolipoprotein E; *APOE4* Apolipoprotein E ɛ4

Data are presented as mean (SD). Groups were compared using an independent *t* test

### DHA incorporation rate (*J*_in_)

The brain incorporation rate of DHA (*J*_in_) was calculated using the global gray matter (average of 19 gray matter regions) value for k* before PVE correction multiplied by plasma unlabeled unesterified DHA concentrations. Unesterified plasma DHA concentrations were not different between *APOE4* noncarriers and *APOE4* carriers (2.0 ± 1.1 vs. 2.2 ± 1.5 nmol/ml, respectively; *p* = 0.76). Among *APOE4* noncarriers and carriers, gray matter *J*_in_ was 5.0 ± 3.3 vs. 6.3 ± 4.5 μmol/day/g, respectively (*p* = 0.49). With a mean whole brain volume of 1242 ml (calculated by MRI), this DHA incorporation rate was equivalent to a daily whole brain DHA incorporation rate of 3.8 ± 2.5 mg/day for *APOE4* noncarriers and 4.6 ± 3.3 mg/day for *APOE4* carriers (*p* = 0.5). Gray matter *J*_in_ was not significantly different between the *APOE* groups, mainly because plasma DHA concentration had large variance in the two groups.

### Cerebral blood flow

Neither mean overall gray matter nor medial temporal lobe CBF differed significantly between the *APOE* subgroups (global gray matter CBF 69.9 (16.7) ml×100 g^-1^ ×minute^-1^ in noncarriers vs. 71.4 (11.8) ml×100 g^-1^ ×minute^-1^ in *APOE4* carriers; *p* = 0.8). Mean gray matter and medial temporal lobe k* did not correlate with the respective rCBF (data not shown).

## Discussion

In this exploratory post hoc analysis, we identified a significantly greater mean global gray matter DHA incorporation coefficient (k*) in *APOE4* carriers compared with noncarriers. This difference was present in several brain regions, including the posterior cingulate cortex and the medial temporal lobe. Within the medial temporal lobe, higher DHA k* was prominent in the entorhinal cortex area. The simplest explanation for the significantly higher values of k* in *APOE4* carriers is an increased incorporation by the brain from circulating unesterified DHA, replacing DHA that is either metabolized to bioactive products or lost to degradation. Given the small sample size and the exploratory nature of this study, these results are proof of concept and require additional validation.

Vandal et al. reported reduced brain DHA levels in older but not younger *APOE4* mice compared with age-matched *APOE*2 TR mice [20]. We recently reported lower CSF DHA levels in older *APOE4* carriers with AD after 18 months of DHA supplementation than in *APOE4* noncarriers [21]. It is possible that the increased k* represents a compensatory mechanism in younger *APOE*4 carriers to cope with increased brain DHA loss and to maintain brain DHA levels. This mechanism might become impaired with aging, predisposing older *APOE*4 carriers to reduced brain DHA levels and increasing the risk for cognitive decline.

It is not possible on the basis of PET images to distinguish the exact metabolite explaining the higher incorporation of DHA in the brain. The equation for calculating k* assumes that all [1-^11^C]-DHA is irreversibly trapped in the brain and that no radioactive metabolite other than [^11^C]-CO_2_ crosses the blood-brain barrier (BBB). This could result from more efficient transport of unesterified DHA across the BBB, increased activation of DHA to DHA-CoA by an acyl-CoA synthetase, greater esterification into brain membrane lipid by an acyltransferase, or decreased hydrolysis by phospholipase A_2_ (PLA_2_) [26]. Any one of these steps could be influenced by *APOE* genotype.

Several factors can alter k*. For example, k* was decreased in mice genetically lacking calcium-independent PLA_2_β VIA [27], but it was increased when plasma and brain DHA concentrations were reduced by chronic dietary ω-3 PUFA deprivation in rats [17] or in subjects with chronic alcoholism during acute withdrawal of alcohol [22]. Moreover, the DHA transport coefficient was decreased with long-term high-DHA dietary consumption [28]. Therefore, differences in habitual intake of DHA may indirectly affect k*. To reduce variation in DHA intake in the present study, participants were instructed to avoid foods high in ω-3 PUFAs (e.g., seafood) 3 days preceding the PET scan, and they were limited to one caffeinated beverage per day. Beginning 24 h before the PET scan, they consumed standardized meals; in addition, they did not eat for 12 h prior to the scan. The differences in plasma DHA levels or DHA dietary intake were not significant by group.

The lower value of k* in the medial temporal cortex is consistent with previous reports for [1-^11^C]- arachidonic acid and with values for rCBF [29, 30]. The data likely reflect the unique architecture of this region, although there is some effect of the PVE correction [24]. We previously reported that lower plasma levels of DHA were significantly associated with lower entorhinal brain volumes in older cognitively healthy adults with increased brain amyloidosis [13]. Higher ω-3 content of red blood cells was also associated with a lower rate of hippocampal atrophy [31]. Atrophy of this brain region predicts progression to AD [32]. Therefore, understanding the mechanisms that influence DHA metabolism in the medial temporal cortex is of particular relevance to AD.

Higher regional k* among this relatively young adult population of *APOE4* carriers may provide one mechanism for increased regional brain activation observed in young adult *APOE4* carriers [33–35]. One report demonstrated differences in myelin structure and gray matter volume in infants carrying the *APOE4* allele [36]. Although *APOE4* is associated with increased risk for memory decline and AD in older adults, several (but not all) studies suggest a behavioral advantage in *APOE4* for younger carriers [37]. For example, in some studies, *APOE4* has been associated with higher IQ scores [38] and a higher education level [39]. Advantageous effects of the *APOE4* allele have also been found for memory-related functions in young animals. Hippocampal long-term potentiation (LTP) was enhanced at a young age in *APOE4* TR mice compared with *APOE4* noncarrier TR mice [40]. This LTP enhancement was age-dependent and disappeared in the adult mice. Mondadori et al. found an association of *APOE4* with better episodic memory compared with *APOE2* and *APOE3* in 340 young, healthy persons [41]. Dennis et al. found enhanced functional connectivity of the medial temporal lobe with the posterior cingulate cortex in young adult *APOE4* carriers [34]. Rusted et al. reported that the *APOE4* in young adults was associated with improved attention and enhanced connectivity [35]. Filippini et al. reported increased default mode network coactivation in *APOE4* carriers relative to noncarriers using resting-state functional MRI [33]. Combined, these findings suggest a state of increased brain activity decades prior to the onset of cognitive decline in *APOE4* carriers. These reports support the “antagonistic pleiotropy” hypothesis in which cognitive advantages in younger adults support higher achievement and greater selection benefits, but may increase susceptibility to brain exhaustion and memory failure with age [42]. In this context, one possible interpretation of the higher k* is that *APOE4* is associated with greater brain DHA loss and greater incorporation of DHA into the brain from plasma. These findings would suggest a beneficial response in cognitive function by increasing DHA consumption in *APOE4* carriers in order to meet the greater metabolic demand for DHA in the brain. Researchers in several epidemiological studies and clinical trials have reported cognitive benefit from increasing DHA consumption in cognitively healthy *APOE4* carriers [43]. This hypothesis merits additional investigation.

The study has several limitations. The sample size was small, and the study was a post hoc analysis of middle-aged, predominantly white adults. We also did not have sufficient participants to examine the effect of age or separate homozygous from heterozygous *APOE4* carriers. DHA incorporation into the brain may not be dependent upon transport of peripheral lipoproteins; thus, our observed differences may be due to *APOE4*-related differences in transport across the BBB, intracellular transport, metabolism, or degradation processes. Unfortunately, data were not available to evaluate these hypotheses.

## Conclusions

To our knowledge, this is the first study describing brain DHA incorporation coefficient in the context of the *APOE4* allele and shows that brain regions implicated in the development of AD have different DHA incorporation coefficients, depending on *APOE* status. These findings support the development of novel DHA uptake imaging modalities such as [^18^F]-DHA to potentially accelerate the application of DHA imaging in clinical research. Knowledge of brain DHA metabolism will enhance understanding of how the *APOE4* allele affects cognitive function and AD risk across the lifespan.

## Abbreviations

AD: Alzheimer’s disease
APOE: Apolipoprotein E
APOE4: Apolipoprotein E ɛ4
BBB: Blood-brain barrier
BMI: Body mass index
CoA: Coenzyme A
CSF: Cerebrospinal fluid
DHA: Docosahexaenoic acid
DHQ: Diet History Questionnaire
HDL-C: High-density lipoprotein cholesterol
*J*_in_: Docosahexaenoic acid uptake rate
*k**: Docosahexaenoic acid uptake coefficient
LDL-C: Low-density lipoprotein cholesterol
LTP: Long-term potentiation
MRI: Magnetic resonance imaging
PET: Positron emission tomography
PLA_2_: Phospholipase A_2_
PUFA: Polyunsaturated fatty acid
PVE: Partial volume error
rCBF: Regional cerebral blood flow
ROI: Region of interest
TR: Targeted replacement
